# Morpho-Geometric Description of the Skulls and Mandibles of Brown Bears (*Ursus arctos*) from the Dancing Bear Belitsa Park

**DOI:** 10.3390/ani14172541

**Published:** 2024-08-31

**Authors:** Iliana Stefanova Ruzhanova-Gospodinova, Silvi Vladova, Tomasz Szara, Faruk Tandir, Ewa Szara, Ebru Eravci Yalin, Ozan Gündemir

**Affiliations:** 1 Department of Anatomy, Physiology and Animal Sciences, University of Forestry, 1797 Sofia, Bulgaria; 2 Faculty of Veterinary Medicine, University of Forestry, 1797 Sofia, Bulgaria; 3 Department of Morphological Sciences, Institute of Veterinary Medicine, Warsaw University of Life Sciences-SGGW, 02-776 Warsaw, Poland; 4 Department of Basic Sciences of Veterinary Medicine, Veterinary Faculty, University of Sarajevo, 71000 Sarajevo, Bosnia and Herzegovina; 5 Division of Agricultural and Environmental Chemistry, Institute of Agriculture, Warsaw University of Life Sciences, Nowoursynowska 159, 02-776 Warsaw, Poland; 6 Department of Surgery, Faculty of Veterinary Medicine, Istanbul University-Cerrahpasa, 34320 Istanbul, Türkiye; 7 Department of Anatomy, Faculty of Veterinary Medicine, Istanbul University-Cerrahpasa, 34320 Istanbul, Türkiye

**Keywords:** carnivore, cranium, shape analysis, veterinary anatomy

## Abstract

**Simple Summary:**

This study explored the differences in skull and mandible shapes between male and female brown bears using advanced 3D imaging techniques. The main goal was to understand how and why these shapes vary, focusing on whether these differences are due to size or sex. The research found that male brown bears have longer and thinner skulls, with notable changes in specific areas like the back of the head and jaw muscles. These differences are likely linked to the larger body size of male bears, which may require stronger muscles and a different skull structure to support their greater weight and chewing needs. The study highlights the importance of these shape variations for the bears’ adaptation to their environment. Understanding these differences can help conserve and manage brown bear populations by providing insights into their physical development and needs. This knowledge is valuable for wildlife biologists, conservationists, and anyone interested in the natural world, offering a clearer picture of how animals adapt over time.

**Abstract:**

The present study aimed to describe the skull and mandibles of the brown bear (*U. arctos*) from the Dancing Bear Belitsa Park using advanced 3D morpho-geometric techniques. The objective was to explore how sexual dimorphism and size influence cranial structures using advanced 3D geometric morphometric methods. Three-dimensional models of the skulls and mandibles of 12 brown bears were used. Differences in skull morphology between male and female brown bears were observed in this study. The male brown bear skull, larger than the female, exhibited a more pronounced extension of the upper part of the nuchal region towards the posterior. Additionally, the posterior part of the frontal region appeared notably thinner in male brown bears compared to females. Analysis of the mandible revealed that the masseteric fossa was more developed in males than females. These shape differences between males and females were found to be influenced by body size. Statistical analyses indicated a significant allometric effect of body size on skull PC1 values, suggesting that giant bears tend to have more elongated skulls. This implies a relationship in which larger individuals exhibit greater cranial elongation. In contrast, mandible PC1 values showed no size-related variation, indicating that mandibular shape is less influenced by overall body size. However, PC2 values in the mandible increased significantly with larger specimens, indicating a larger masseteric fossa in larger bears. This morphological adaptation potentially enhances feeding efficiency and bite strength in larger individuals, reflecting functional adaptations in brown-bear mandibular morphology. These findings contribute to our understanding of sexual dimorphism and size-related morphological adaptations in brown bears, providing insights into their adaptation biology and ecological roles within their habitats.

## 1. Introduction

Bears evolved within the *Carnivora* clade during the late Oligocene and early Miocene, approximately 20–25 million years ago [1]. The family *Ursidae* comprises eight extant species, which are classified into three subfamilies: *Ursinae* (including the polar bear *Ursus maritimus* and the brown bear *Ursus arctos* (*U. arctos*), the American black bear *Ursus americanus*, the Asiatic black bear *Ursus thibetanus*, the sloth bear *Melursus ursinus*, the Malayan sun bear (*Helarctos malayanus*), *Ailuropodinae* (with the giant panda, *Ailuropoda melanoleuca*), and *Tremarctinae* (with the Andean bear *Tremarctos ornatus*) [2]. Morphologically and taxonomically, bears exhibit the defining traits of carnivores. They possess notably large skulls, proportional to their robust bodies, adapted for solid and effective chewing [3]. 

Morphometric analysis involves understanding shapes through precise measurement methods and determining their differences using statistical techniques [4,5]. Geometric morphometric analysis specifically focuses on analyzing the positions of anatomical points in Cartesian coordinates and the homologous points [6]. This method can be applied to any object, whether living or non-living, having a specific shape [7,8,9]. Geometric morphometrics provides a powerful tool for capturing and analyzing the complex shapes of biological structures [10]. Using landmarks, specific points reliably located across specimens allow for quantifying shape variation and comparison across individuals or groups [11,12]. The primary advantage of this technique is its ability to retain geometric information about the studied structures, making it possible to visualize shape differences and deformations with high precision. The skull, in particular, is an ideal subject for geometric morphometric analysis, for several reasons [13]. First, the skull is highly complex, with numerous anatomical landmarks that can be consistently identified across individuals. This makes it possible to capture detailed shape information. Furthermore, evolutionarily and functionally, the skull is one of the most significant parts of the skeleton, reflecting adaptations related to feeding, sensory functions, and brain protection [14,15]. Studying skull morphology can provide insights into species’ evolutionary relationships, functional adaptations, and ecological roles.

In recent years, geometric morphometric studies of the skull have become increasingly common in the literature, especially in animals, as it is one of the bones that best reflects species’ characteristics [16,17,18,19,20]. These studies have utilized 3D geometric morphometry to explore various aspects of skull shape, including allometric effects, sexual dimorphism, and phylogenetic relationships [21,22]. Three-dimensional models, in particular, enhance the accuracy and depth of morphometric analysis, allowing for a more comprehensive understanding of shape variation [23,24]. In this study, we employed 3D geometric morphometry to examine shape variations in the skulls of brown bears (*U. arctos*). This approach enabled us to capture and analyze detailed shape information, providing insights into these animals’ morphological differences and adaptations [25]. Three-dimensional models and advanced morphometric techniques allowed us to explore inter- and intra-group variations, contributing to our understanding of how genetic diversity influences skull morphology in brown bears.

The order Carnivora is one of the most ecologically diverse mammalian lineages, encompassing cats, dogs, bears, and ferrets, each exhibiting a wide range of skull shapes and sizes [26]. This skull shape diversity results from various habitat and behavioral pressures, with diet being the most significant factor [26]. In Carnivora, skull shapes have evolved to suit different feeding habits [27]. The bear family, Ursidae, also shows distinct cranial shape variations influenced by these factors. For example, polar bears have a reduced surface area of the grinding molar teeth, a feature more pronounced in omnivorous ursids, and a low, slender skull [27,28]. This illustrates the notable correlation between feeding ecology and cranial morphology within the Ursidae family [29,30]. Understanding the shape of the mandible and skull provides insights into the environmental conditions and lifestyles associated with feeding, and reveals developmental adaptations [29]. For instance, research has demonstrated that current Ursus genus members’ mandibular and cranial morphology reflects a shift from carnivorous to herbivorous dietary tendencies [31,32]. This shift highlights how dietary needs have driven evolutionary changes in skull structure, offering valuable information about their adaptive strategies over time.

Different anatomical features in living organisms can reveal their evolutionary adaptations, which are influenced by their dietary needs and ecological roles [33,34]. For instance, wild animals’ skulls and jaw bones are often more robust and adapted for tearing apart prey [27]. In contrast, these structures are less developed in domesticated animals due to long adaptation periods to a less-demanding diet. Since 2000, Belitsa Park in Bulgaria has been a center providing protection and care for various brown bears living in poor captivity. Having lived in captivity away from the wild, these bears may exhibit unique structural features, due to their living conditions. 

The present study aimed to describe the skull and mandibles of the brown bear (*U. arctos*) from the Dancing Bear Belitsa Park using advanced 3D morpho-geometric techniques. Additionally, size differences between female and male bears were analyzed. Consequently, this study aims to provide valuable insights into the evolutionary adaptations and ecological roles reflected in the skull morphology of brown bears.

## 2. Materials and Methods

### 2.1. Skulls and Mandibles

The skulls of 12 brown bears (*U. arctos*) from the Dancing Bear Belitsa Park in Bulgaria, belonging to the University of Forestry, Faculty of Veterinary Medicine, were used for this study. All but one of the samples’ ages and approximate weights were obtained from the Park’s records. Detailed information about the samples is provided in Table 1.

### 2.2. Modeling

The skulls and mandibles from the 12 brown bear (*U. arctos*) samples were meticulously scanned to create 3D models using the Shining 3D EinScan SE V2 3D scanner (Shining 3D, Hangzhou, China). This process involved fixed scanning using a rotary table to ensure comprehensive coverage of each specimen. A dot interval of 0.1 mm was employed to capture fine details, and the scanning speed was set at 14 frames per second (FPS).

After the scanning procedure, the raw data were processed using EXScan software (Shining 3D, Hangzhou, China, v3.1.0.0) designed to handle high-resolution 3D scans. This software facilitated the creation of mesh models by connecting the captured points into a continuous surface representation of the skulls and mandibles. The final 3D models were then saved in PLY format, a widely used format for storing 3D data that includes geometric information.

### 2.3. Data

The study employed 3D geometric morphometric analysis, beginning with developing an initial set of landmarks. This process utilized the PseudoLMGenerator module within the Slicer program (version 5.2.2) [35]. A plane was used for both the skull and mandible to ensure that the points were evenly distributed on both sides of the bone samples.

**Figure 1:**
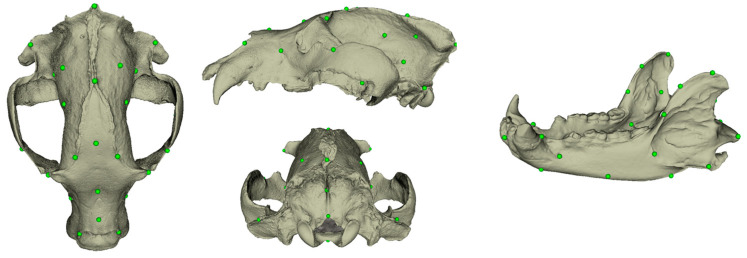
Landmarks.

A 10% tolerance range was applied during the draft landmark process, ensuring that the landmarks were appropriately positioned and any minor discrepancies accounted for. As a result of these procedures, draft sets of landmarks were established, consisting of 32 landmarks for the skull and 26 landmarks for the mandible (Figure 1).

In this study, researchers utilized Automated Landmarking through Point Cloud Alignment and Correspondence Analysis (ALPACA) to apply the initial draft landmark set across all other 3D models [36]. This technique, integrated within the ALPACA module in the Slicer program (version 5.2.2), enabled the automatic application of the draft landmark set to all specimens. This automation significantly improved the efficiency of the landmarking process, while maintaining uniformity throughout the entire dataset [35].

The ALPACA method works by aligning the point clouds of the 3D models and pinpointing corresponding points across these models. By automating the landmarking process, ALPACA minimizes human error and streamlines the analysis of extensive datasets. The process begins by superimposing the 3D models to ensure that analogous anatomical features are closely matched. This alignment is essential for accurately identifying homologous points across different specimens. ALPACA identifies corresponding points following the alignment, ensuring that landmarks are consistently placed in equivalent anatomical positions on each model. This automated process is highly efficient, significantly reducing the time required for manual landmarking while ensuring high accuracy and reproducibility.

### 2.4. Statistical Analysis 

Centroid-size data were gathered for each brown bear sample, providing quantitative insights into their overall size variations. These data were pivotal in investigating general morphological differences across the samples and specific differences between male and female brown bears. The differences in size between sexes were rigorously examined using ANOVA (Analysis of Variance).

Principal Component Analysis (PCA) was employed to explore the shape variations in the skulls and mandibles of the brown bears. PCA identifies patterns in complex datasets by transforming correlated variables (landmark coordinates in this case) into a set of orthogonal components called principal components (PCs). PC1 and PC2 were identified as components explaining the highest variation in the brown bear skull and mandible morphology. Visual representations, such as graphs, were used to illustrate how individuals were distributed along PC1 and PC2, providing insights into the primary patterns of morphological variation within the sample. Furthermore, the study visualized how variations in PC values influence the morphology of brown bear skulls and mandibles. This visual analysis helps interpret how specific PCs correlate with distinct morphological features or changes, enhancing our understanding of shape diversity among the specimens.

Multivariate regression analysis was conducted to investigate the relationship between size and shape. This analysis specifically examined how centroid size relates to PC1 values, which are associated with brown bears’ most significant shape variation.

## 3. Results

### 3.1. Size

**Figure 2:**
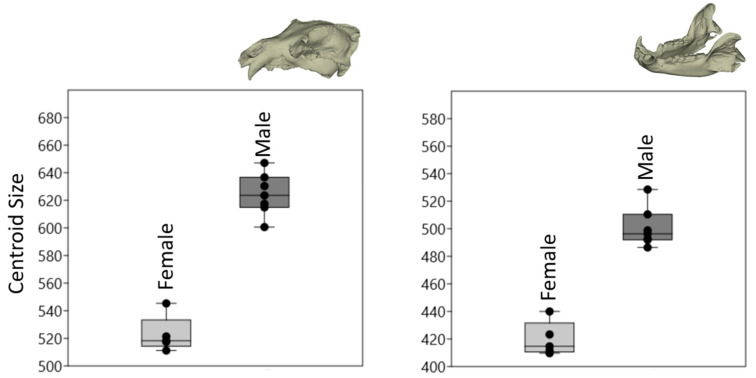
Boxplot with variation in centroid-size values. The darker horizontal line is the median, the margins of the boxes represent the percentiles (25 and 75), and the extensions of the bars represent the maximal and minimal values.

Figure 2 presents centroid-size values for both the mandible and skull of male and female brown bears. It was observed that male individuals had larger skulls and mandibles, correlating with their body weights.

Statistical analysis confirmed this anticipated size difference between sexes to be highly significant (*p* < 0.001). This finding underscores the robustness of the centroid-size measurements in distinguishing size variations between male and female brown bears.

### 3.2. Shape Variation

**Figure 3:**
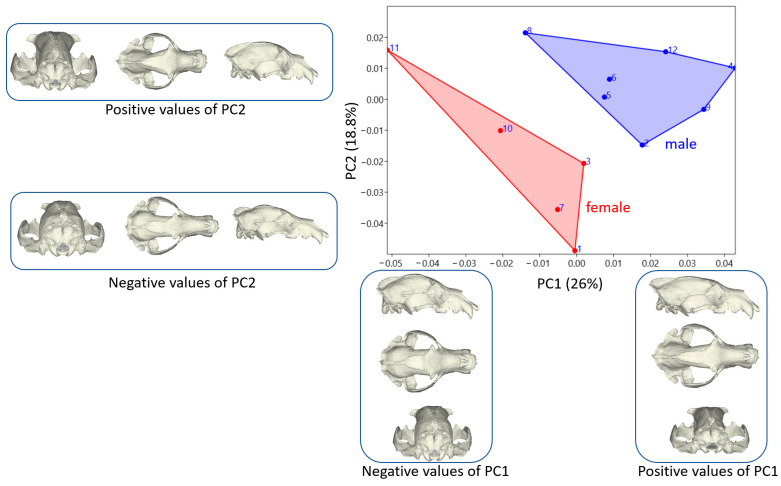
Principal component analysis scatter plot for the skull. Models describing how skull shape changes between the negative and positive values of PC1 and PC2 (Blue: Male; Red: Female).

PC1 accounted for 26% of the total variation in skull morphology, while PC2 explained 18.8%. The distribution of individuals across these two principal components is illustrated in Figure 3.

Male brown bear skulls exhibited positive mean values for PC1 and PC2, indicating a specific shape variation distinct from that of female individuals, who showed negative variation in both components. PC1 notably contributed to separating samples by sex, with nearly all male individuals clustering separately, except for one. PC2, while generally distinguishing between sexes, showed some overlap, notably with two female samples displaying shape variations similar to males. These findings highlight how PC1 predominantly delineates sexual dimorphism in skull shape among brown bears. At the same time, PC2 introduces additional nuances in morphological variation which, albeit generally distinctive, may exhibit overlaps in specific instances.

PC1, which accounted for the highest variation in skull morphology, primarily influenced the nuchal region. At positive PC1 values, the upper part of the nuchal region extended further caudally, exceeding the posterior border of the skull. This resulted in a noticeable elongation and narrowing of the neurocranium.

The most prominent morphological change identified by PC2 was in the height of the skull. Higher PC2 values corresponded to a higher skull shape, while negative PC2 values indicated a lower skull height. Additionally, at positive PC2 values, the posterior part of the frontal bone exhibited a more pointed shape, contrasting with the oval shape observed at negative PC2 values. Furthermore, increasing PC2 values led to a narrower nape region at the back of the skull.

Analyzing these morphological changes and the distribution of PC values provides insights into the distinct features of male brown bears’ skulls. Male individuals tend to have a nuchal region that extends further caudally, beyond the head’s nape. Their neurocranium tends to be elongated and slender, unlike the more oval shape typically seen in female brown bears. Moreover, the posterior part of the frontal region is notably thinner in male brown bears compared to females.

**Figure 4:**
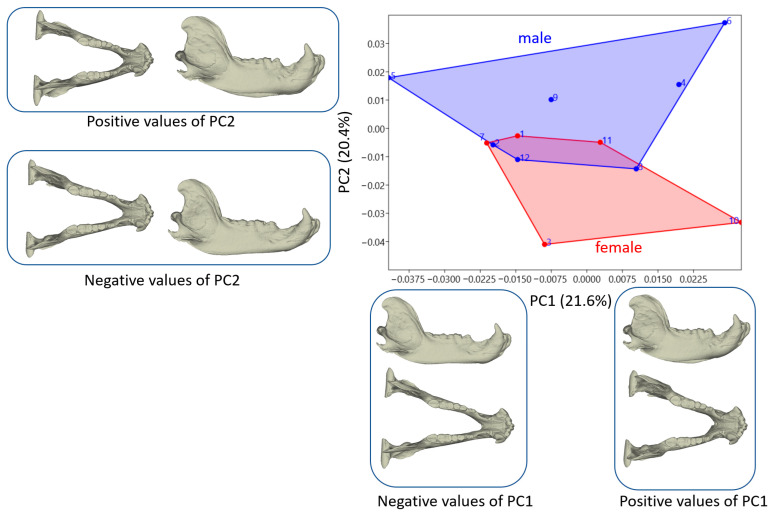
Principal component analysis scatter plot for the mandible. Models describing how mandible shape changes between the negative and positive values of PC1 and PC2. (Blue: Male; Red: Female).

PC1 explained 21.6% of the total variation in mandible morphology, highlighting its significant influence. Meanwhile, PC2 accounted for 20.4% of the variation, contributing notably to the observed morphological diversity. Figure 4 depicts the distribution of individuals along these principal components. In contrast to the skull results, mandible PC1 values showed that shape variations between male and female individuals were relatively similar. Male brown bears exhibited a more comprehensive range of shape variations based on PC1 values. Regarding PC2, while males displayed positive mean values and females showed negative mean values, the difference in shape variations was not statistically significant.

The most significant shape change observed in the mandible was along the ventral edge. At positive PC1 values, the lower edge of the mandible appeared more oval, whereas at negative PC1 values, it exhibited a straighter profile. PC2 revealed the most notable shape change in the masseteric fossa. A positive PC2 value indicated a larger masseteric fossa in shape. Male brown bears exhibited higher PC2 values on average, suggesting a broader masseteric fossa than females.

### 3.3. Allometry

**Figure 5:**
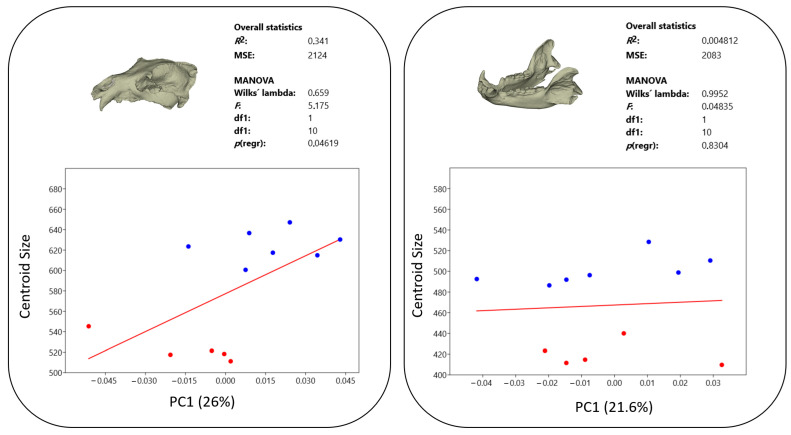
Size effect on PC1 for skull and mandible. (Blue: Male; Red: Female).

The allometric effect of size on both skull and mandible PC1 values was investigated, and the results are presented in Figure 5. A statistically significant positive regression was observed between size and PC1 values in the skull. As size increased, there was a trend towards the upper part of the nuchal region extending further caudally, surpassing the posterior border of the skull. This led to a distinct elongation and narrowing of the neurocranium. However, the relationship between mandible size and PC1 value was statistically insignificant. This suggests that mandible shape variation captured by PC1 is not strongly influenced by overall mandible size in the brown bear population studied. However, the effect of size on PC2 in the mandible was statistically significant (*p* ≤ 0.05). As size increased, there was a corresponding increase in PC2 values. This relationship suggests larger individuals had larger masseteric fossa, highlighting a size-dependent variation in mandibular morphology within the studied brown bear population.

## 4. Discussion

The results of this study reveal significant sexual dimorphism in the skull and mandible morphology of brown bears (*U. arctos*), emphasizing both size-related and sex-specific shape variations. Male brown bears, more extensively than females, exhibited distinct cranial characteristics, such as a more elongated upper nuchal region and a thinner posterior frontal bone. These differences suggest possible effects related to body size. For example, the variations observed in the masseteric fossa of the mandible, where males with larger structures exhibit more developed features than females, may represent functional adaptations related to bite force and chewing efficiency. This size difference in mandibular morphology may reflect the need for increased mechanical advantage during feeding, particularly in larger males. Statistical analyses underscored a significant allometric effect of body size on skull PC1 values, indicating that larger individuals tend to have more elongated skulls. This finding aligns with theories of adaptive morphology in larger-bodied mammals, where structural elongation may enhance biomechanical advantages for activities such as feeding or interspecies competition [37,38]. Conversely, mandible PC1 values did not exhibit size-related variation, suggesting that mandibular shape may be more conserved across size categories within the studied brown bear population. However, PC2 values in the mandible significantly increased with larger specimens, indicating a proportionally larger masseteric fossa. This adaptation likely enhances the mechanical efficiency of chewing in more giant brown bears, reflecting an adaptive response to dietary and ecological pressures.

In the study, it was observed that male brown bear skulls exhibited different shape variations compared to those of females. However, these variations might be influenced by differences in size rather than sex. Male individuals had significantly larger body mass than female individuals, which could lead to morphological differences in the skull. The most notable feature was the backward sagging of the nuchal region in male skulls, a trait likely related to their greater size. The nuchal region is an attachment point for several muscles supporting the skull. During the adaptation process, the increased skull weight in males may have caused morphological changes in these muscle attachment areas. This adaptation would be essential for maintaining balance and stability in more giant skulls. The observed backward projection of the nuchal region in males indicates that these areas have adapted to support more substantial muscle attachments necessary for supporting a heavier skull. Additionally, the increased chewing force, which tends to develop in proportion to size, may have contributed to morphological changes in the temporal region of the skull. This could result in more lateral pressure on the skull, producing a thinner and longer skull shape in males [39]. This idea is supported by the PC2 results of the mandible, where male individuals had a wider masseteric fossa compared to females. The masseteric fossa is a crucial area for muscle attachment, which influences chewing efficiency. A larger masseteric fossa in males suggests that their skull morphology has adapted to accommodate stronger and more efficient chewing muscles. However, to scientifically determine whether these differences between male and female brown bears are due to sexual dimorphism or allometric changes, it would be necessary to compare samples of the same weight. This comparison would help to isolate the effects of size from those of sex. If morphological differences persisted even when size is controlled, this would support the idea of sexual dimorphism. Conversely, if the differences diminished when size is accounted for, it would indicate that the observed variations are primarily due to allometric changes. Overall, the study highlights the importance of considering both size and sex when examining morphological variations in brown bear skulls. The findings suggest that the shape differences observed between male and female skulls are likely a combination of sexual dimorphism and allometric adaptation to size. Further research, including comparisons of weight-matched samples, is needed to disentangle these effects and provide a more precise understanding of the factors driving skull morphology in brown bears.

Using geometric morphometric techniques has been instrumental in revealing shape variations in sexually dimorphic structures across various species, such as the pelvic bone in humans or the carapace in turtles [40,41]. These studies have consistently demonstrated distinct shape differences between males and females. Interestingly, some earlier skull studies reported that sexual dimorphism was not statistically significant, suggesting limited sex-based effects on skull morphology [42]. However, research specifically focused on bears, such as Ghanbari’s study [43] on Iranian brown bears, has consistently highlighted pronounced sexual dimorphism in skull shape. Ghanbari’s findings indicated significant differences in the occipital crest, zygomatic arc, parietal bones, and dental rows between male and female brown-bear skulls. Similarly, Nezami [44], in another study from Iran, reinforced these observations by identifying significant variations in skull shape between male and female brown bears. They emphasized the structural reinforcement and strength differences evident in male skulls compared to females. In the current study on brown bears, while shape differences between male and female skulls were observed, it is essential to consider the potential influence of the size differences mentioned earlier. This aligns with the understanding that variations in skull morphology could be attributed to sexual dimorphism and allometric factors driven by size differences between sexes. Further investigation, particularly comparing samples matched for body size, could clarify the relative contributions of sexual dimorphism and allometric growth patterns to the observed shape variations in brown bear skulls.

Sexual dimorphism of the skull is marked in different systematic groups of mammals. It is most pronounced in ruminants, related to the weapons used in mating fights [45,46]. In carnivores, bite characteristics have been noted as differing between sexes. Gittleman and Valkenburgh [47] found that male ermine canines (Mustela erminea) are almost twice as large as those in females. Forbes et al. [48] indicated that male red foxes (Vulpes vulpes) have proportionally longer and wider skulls, with a greater zygomatic arch width and longer temporal fossa (sagittal crest), due to their larger skulls. A study on brown bears yielded similar findings. The neurocranium of male brown bears tends to be elongated and slender, contrasting with the more oval shape typically seen in females. However, this shape difference may be attributed to size, as seen in allometry results. Increased size may affect bite force, which influences shape variations, depending on the biting muscles. Furthermore, a study on sexual dimorphism in carnivore skulls found that the evolution of sexual dimorphism in bite force is more closely related to cranial size than cranial shape [49].

The animals used in the study were obtained from the Dancing Bear Belitsa Park. These animals have spent a significant portion of their lives in captivity, so their dietary habits may have altered at some point, compared to their natural wild counterparts. Previously kept as pets, most of these bears have diverged from their wild-diet cycles, which may have led to lifestyle changes that could influence skull and lower-jaw morphologies. Therefore, these factors should be carefully considered when comparing the results of this study with other brown bears living in the wild. Additionally, it is essential to recognize that diet and the different social behaviors exhibited in captivity could contribute to specific morphological adaptations.

## 5. Conclusions

In conclusion, this study employing geometric morphometric analysis has elucidated significant shape differences in the skulls of male and female brown bears. The observed variations underscore robust sexual dimorphism in skull shape among brown bears. These findings align with previous research highlighting similar patterns in other bear species. Moreover, the influence of body size on these shape differences suggests a complex interplay between sexual dimorphism and allometric growth patterns. 

## Figures and Tables

**Table 1:** Samples used in the study, their sex, age, and estimated weight.

Samples	Sex	Age	Weight (kg, Approximately)
1	Female	39	100
2	Male	34	250
3	Female	30	150
4	Male	29	300
5	Male	32	200
6	Male	31	300
7	Female	37	120
8	Male	33	206
9	Male	36	200
10	Female	37	100
11	Female	34	100
12	Male	Unknown	Unknown

## Data Availability

The data presented in this study are available upon request from the corresponding author.
